# Caffeinated chewing gum produces comparable strength and power gains to capsules with fewer side effects in resistance-trained men

**DOI:** 10.1080/15502783.2025.2531173

**Published:** 2025-07-09

**Authors:** Li Ding, Jue Liu, Yixuan Ma, Litian Bai, Li Guo, Bin Chen, Yinhang Cao, Olivier Girard

**Affiliations:** a Shanghai University of Sport, Key Laboratory of Exercise and Health Sciences (Ministry of Education)School of Athletic Performance, Shanghai, China; b Shanghai University of Sport, School of Athletic Performance, Shanghai, China; c Fudan University, Department of Rehabilitation Medicine, Huashan Hospital, Shanghai, China; d Shanghai University of Sport, School of Exercise and Health, Shanghai, China; e Fujian Agriculture and Forestry University, Department of Public Physical Education, Fuzhou, China; f The University of Western Australia, School of Human Sciences (Exercise and Sport Science), Perth, Australia

**Keywords:** Ergogenic aid, caffeine administration, one-maximum repetition, resistance exercise, load-power relationship

## Abstract

**Background:**

Caffeine, widely used as an ergogenic aid, has been extensively studied regarding its dosage and timing of ingestion. However, the impact of different administration methods on caffeine’s performance-enhancing effects remains relatively underexplored. This study compared the effects of caffeine administered via chewing gum versus capsules on maximal strength, muscular power, and side effects during bench press and back squat exercises.

**Methods:**

Sixteen resistance-trained males participated in a double-blind, randomized trial, ingesting either a 4 mg/kg caffeine capsule (CC) or placebo capsule (PC) one hour before testing, or a 4 mg/kg caffeinated gum (CG) (4 mg/kg) or placebo gum (PG) five minutes prior. Assessments including one-repetition maximum (1RM) and muscular power at 25%, 50%, 75%, and 90%1RM for bench press and back squat.

**Results:**

Caffeine increased 1RM (+2.1–5.0%) and muscular power (+6.1–20.0%) in both the bench press and back squat compared to placebo (all *p* < 0.05). However, no significant differences were observed between CC and CG for maximal strength or muscular power (all *p* > 0.05). Furthermore, CG was associated with fewer reports of gastrointestinal discomfort (12.5% vs. 37.5%) immediately post-exercise and tachycardia/heart palpitations (0% vs. 25.0%) at 24 hours compared to CC (all *p* < 0.05).

**Conclusion:**

Caffeinated gum (4 mg/kg) produced ergogenic effects comparable to capsules in enhancing maximal strength and muscular power during bench press and back squat exercises, with fewer side effects in resistance-trained men.

## 1. Introduction

Caffeine (1,3,7-trimethylxanthine) is widely consumed in food and beverages [1], and is one of the most commonly used substance in sports. It has been shown to enhance performance in endurance activities [2], anaerobic efforts [3], and strength and power-based tasks [4]. For instance, caffeine supplementation (6 mg/kg, capsule) increased eccentric strength and power of the knee flexors and extensors, measured with isokinetic dynamometry and countermovement jumps, in female team-sport players both during a 90-min intermittent running protocol and again the following morning [5]. When administrating caffeine, several factors must be considered to maximize its ergogenic benefits while minimizing side effects (i.e. individual sensitivity) [6]. While dosage and timing of ingestion have been widely studied [2,7,8], the method of administration method remains relatively under explored.

Among various administration methods (e.g. capsules, gum, strips, gels, and mouthwash), capsules and chewing gums are the most popular [9–11]. Studies have demonstrated that caffeine (4–6 mg/kg) in capsule form significantly enhances maximal strength (one-repetition maximum [1RM]) [12,13] and muscular power (barbell velocity and/or power output) [14,15] during bench press and back squat in resistance-trained individuals. Caffeine capsules (CC) typically reach their maximal effectiveness on muscular strength within 30–60 min after ingestion, coinciding with the time needed to achieve peak plasma concentrations [16]. Alternatively, caffeine delivered via chewing gum is absorbed more rapidly through the oral mucosa, resulting in faster onset of action (5–10 min *versus* ~60 min for capsules) [17–19]. This rapid absorption has generated growing interest in the effects of caffeinated gum (CG) on resistance exercise performance.

Research using CG (3–3.6 mg/kg) has shown improvements in maximal strength and muscular power in the bench press and back squat. One study from our group showed increased 1RM for both exercises [20], while another reported higher barbell mean velocity at 50–90%1RM in the bench press among resistance-trained men [21]. Indirect comparisons indicate that CG may elicit greater improvements in maximal strength (+5.0–6.8% vs. +1.3–4.5%) and muscular power (+5.7–9.1% vs. +1.0–5.6%) compared to capsules [12–15]. This may be partly explained by CG’s ability to bypass first-pass metabolism, leading to faster absorption and fewer side effects (e.g. *gastrointestinal discomfort* and *tachycardia and heart palpitations*) [20]. However, no study has yet directly compared the effects of these two caffeine administration methods on maximal strength, muscular power, and side effects within the same cohort of resistance-trained individuals during bench press and back squat exercises.

This study aimed to compare the ergogenic effects of caffeine (4 mg/kg) administered through chewing gum *versus* capsules on maximal strength and muscular power during the bench press and back squat, and the incidence of related side effects. We hypothesized that caffeine from chewing gum will have greater benefits on maximal strength and muscular power during these exercises than capsules, with fewer side effects.

## 2. Methods

### 2.1. Participants

Sample size estimation was performed using an *a priori* power analysis with G*Power (version 3.1; Universität Düsseldorf, Düsseldorf, Germany), based on an α-level of 0.05, a 1-β error probability of 0.8, a correlation coefficient of 0.9 [21], and effect sizes (*f*) ranging from 0.18 to 1.28 for maximal strength and muscular power during bench press and back squat exercises [14,20,22]. The analysis revealed that a sample of 10 participants would be sufficient to detect the effect of caffeine on 1RM and muscular power. To account for potential dropouts, 16 participants were ultimately recruited (mean ± standard deviation [SD]: age 20.8 ± 1.6 yr, height 173.5 ± 5.1 cm, body mass 72.9 ± 6.1 kg, fat body mass 11.2 ± 3.0 kg, lean body mass 61.7 ± 5.3 kg, training history 4.2 ± 1.9 yr, 1RM bench press 92.0 ± 12.4 kg, 1RM back squat 152.5 ± 20.6 kg). Habitual caffeine intake (41.4 ± 45.1 mg/day) was assessed using a validated self-reported questionnaire [23], and all participants were categorized as either caffeine-naive or mild caffeine consumers (0–2.99 mg/kg/day) [24]. None of the participants withdrew during the entire experiment.

The inclusion/exclusion criteria were: (1) healthy men aged 18–35 years; (2) at least two years of resistance training experience, with a minimum of three sessions per week during the three months preceding the experiment; (3) ability to perform the bench press and back squat with loads of at least 100% and 125% of body mass, respectively ; and (4) no self-reported smoking or caffeine allergy. All participants provided written informed consent. This study was approved by the Scientific Research Ethics Committee of Shanghai University of Sport (No. 102772023RT013).

### 2.2. Experimental design

**Figure 1.**
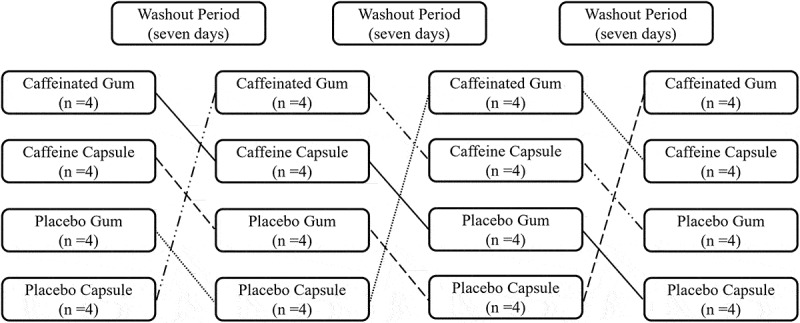
Latin square design in four experimental sessions.

This experiment employed a double-blind, placebo-controlled, crossover, and randomized design. Participants attended five laboratory sessions: one for familiarization and 1RM assessments (bench press and back squat), and four times for experimental sessions. In each trial, participants were randomly assigned to ingest either a 4 mg/kg CC or placebo capsule (PC) one hour prior, or chew 4 mg/kg CG or placebo gum (PG) for 5 min before performing maximal strength (1RM) and muscular power assessments at incremental loadings (25%, 50%, 75%, and 90%1RM) for both bench press and back squat. To minimize potential order effects, a 4 × 4 Latin square design was utilized for randomization and counterbalancing [25] (Figure 1). All visits were separated by seven days to ensure full recovery and prevent learning effects [4], and they were scheduled between 12:00 and 16:00 to control for circadian influences [13]. Participants were required to avoid intense exercise and caffeine (e.g. coffee, chocolate, soda, and energy drinks) for 24 hours prior to each visit.

### 2.3. Experimental protocol

During the familiarization session, participants’ height, body weight and body composition were measured using electric bioimpedance (X-scan Plus II, Jawon, Korea). One experienced personal trainer then assessed each participant’s 1RM for the bench press and back squat using free-weight equipment (Cybex, Medway, MA, USA), following the guidelines of Baechle and Earle [26]. The 1RM values were used to determine individualized loads (25%, 50%, 75%, and 90%1RM) for both exercises. Participants’ dietary and physical activity habits from the preceding 24 hours were recorded using a 24-hour recall questionnaire and the International Physical Activity Questionnaire (IPAQ) [27], and these habits were maintained consistently throughout subsequent sessions.

**Figure 2.**
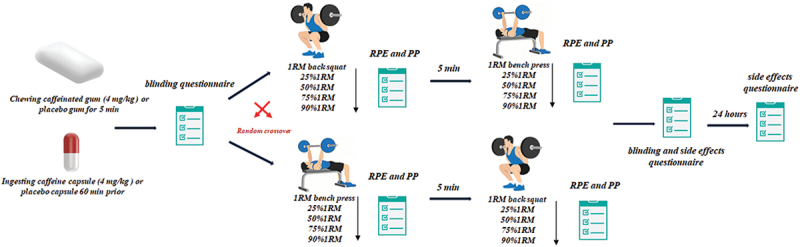
Overview of the experimental protocol.

During the experimental sessions, participants ingested either CC, CG, PC or PG before starting the tests (Figure 2). The protocol began with a 1RM measurement, followed by assessments of muscular power at incremental loads (25%, 50%, 75%, and 90%1RM) during both bench press and back squat. The exercise order was counterbalanced across participants and remained consistent for each individual in both sessions, using randomization software (Excel, Microsoft, Washington, USA). Two experienced personal trainers supervised the exercises to ensure proper technique [26]. Participants were also prohibited from wearing weightlifting belts, bench shirts, or other supportive garments during the tests. Furthermore, at each visit, participants’ 24-hour caffeine intake was recorded using a validated self-reported questionnaire [23], which revealed that that all participants strictly adhered to caffeine withdrawal.

#### 2.3.1. Maximal strength (1RM) test

Prior to the 1RM test, participants performed a warm-up consisting of 10 and 5 repetitions at 50% and 75%1RM, respectively, as assessed during the familiarization session [7]. Following the recommendations of Baechle and Earle [26], the 1RM was determined within 3 to 5 attempts, with 5-min rest periods between successful attempts. Ratings of perceived exertion (RPE) and pain perception were assessed within 5 seconds after each successful attempt [28,29]. Pilot testing in our laboratory showed *excellent* interclass correlation coefficients (ICC) for 1RM measurements for the bench press (ICC = 0.98, 95% CI = 0.96–0.99) and back squat (ICC = 0.98, 95% CI = 0.95–0.99) across three days.

#### 2.3.2. Muscular power test

After a 5-min rest following 1RM determination, participants performed the same exercises at 25%, 50%, 75%, and 90% of their 1RM (as measured during the familiarization session), using a 2/0/X/0 cadence (2 seconds eccentric phase, no pause at transition, X representing maximum concentric tempo, and 0 seconds at the end of the movement) [30,31], stabilized by a metronome set to 30 beats/min. Participants completed three repetitions at 25%1RM, two repetitions at 50%1RM, and one repetition at 75% and 90%1RM, with 3-min rest intervals between sets. The GymAware Power Testing system (Kinetic Performance Technologies, Canberra, Australia), which has demonstrated excellent reliability in measuring velocity and power output [32], was used to record bar displacement during the concentric phase, including mean velocity (MV in m/s), peak velocity (PV in m/s), mean power output (MPO in W), and peak power output (PPO in W). The 6–20 point RPE scale and the 0–10 point pain perception scale (45, 46) were used to assess the participants’ RPE and pain perception within 5 seconds following the final repetition. Pilot testing in our laboratory indicated *excellent* interclass ICC for MV (bench press: ICC = 0.89, 95%CI = 0.82–0.93; back squat: ICC = 0.89, 95% CI = 0.82–0.94) and MPO (bench press: ICC = 0.96, 95% CI = 0.94–0.98; back squat: ICC = 0.94, 95% CI = 0.91–0.97) across seven testing days.

#### 2.3.3. Supplementation protocol

A 3–6 mg/kg caffeine dose is considered optimal for enhancing performance with minimal side effects [2]. However, as 3 mg/kg is insufficient to improve muscular power at 75–90% 1RM [22] and 6 mg/kg requires chewing 4–6 pieces of gum – impractical for full mastication – a dose of 4 mg/kg was selected for this study. Caffeine was administered in two forms (capsule or gum), with each administration mode paired with a corresponding placebo. For the capsule condition, CC and PC were ingested 1 hour before testing. The capsules contained either caffeine powder (Sigma-Aldrich, Sydney, USA) or dextrose as a placebo. For the gum condition, participants chewed the gum (CG and PG) for 5 min before testing, then disposed of it in a designated container. Previous research confirmed that 5 min of chewing releases approximately 85% of the caffeine content (19). The CG and PG were commercially available Military Energy Gum (Market Right Inc., Plano, IL, USA) and non-caffeinated chewing gum (Spearmint Extra Professional; Wrigley’s, Chicago, IL, USA), respectively. All samples were individually calculated based on each participant’s body mass and weighted using a high-precision digital scale (accuracy: ±0.001 mg). All individuals, except the designated experimenters, were blinded to the sample in terms of taste, shape, and size.

#### 2.3.4. Assessment of blinding and side effects

To evaluate blinding effectiveness, participants were asked, *“What substance do you think you ingested?”* immediately after ingesting caffeine and post-exercise. The response options were: (a) caffeine; (b) placebo, or (c) I don’t know [33]. Participants also completed a Side Effects Questionnaire, a nine-item dichotomous scale, to evaluate potential caffeine side effects immediately after exercise and 24 hours later [34].

### 2.4. Statistical analysis

Data were analyzed using SPSS (Version 22.0; IBM Corp., Armonk, NY, USA) and reported as mean ± standard deviation (SD), with statistical significance set at *p* < 0.05. The Shapiro-Wilk test was used to assess the normal distribution of all variables. A two-way repeated measures ANOVA (condition [caffeine and placebo] × mode [capsule and gum]) was used to compare performance (1RM) and subjective responses (RPE and pain perception) during the maximal strength test. A paired sample t-test was employed to evaluate percentage changes in 1RM between modes. A three-way repeated measures ANOVA (load [25%, 50%, 75%, and 90%1RM] × condition [caffeine and placebo] × mode [capsule and gum]) was used to examine differences in performance (PV, MV, MPO, and PPO) and subjective responses (RPE and pain perception) during the muscular power test. A two-way repeated measures ANOVA (load [25%, 50%, 75%, and 90%1RM] × mode [capsule and gum]) was used to examine differences in the percentage change in muscular power. Partial eta squared (*ηp^2^*) values were calculated to estimate effect sizes for main effects. Significant main effects were followed by *Bonferroni* correction for post hoc testing. Cohen’s *d* was calculated for pairwise comparisons to assess effect sizes, categorized as *trivial* ( < 0.20), *small* (0.20–0.49), *moderate* (0.50–0.79), and *large* (≥0.80) (58). Additionally, the Bang’s Blinding Index (BBI) was used to assess blinding effectiveness, and Cochran’s Q test was applied to detect variations in side effects between treatments (CC, PC, CG, and PG).

## 3. Results

### 3.1. Muscular strength

**Figure 3.**
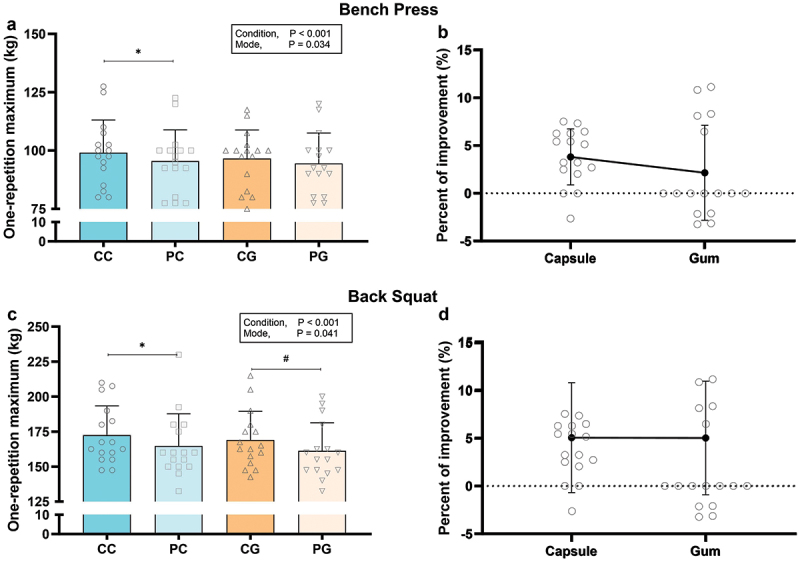
One-repetition maximum and percentage improvement in the bench press (a and b) and back squat (c and d) exercises.

A significant main effect of condition was detected for 1RM in both the bench press (*F* = 28.256, *p* < 0.001, *ηp^2^* = 0.65) and back squat (*F* = 18.491, *p* < 0.001, *ηp^2^* = 0.55) (Figure 3). Post-hoc tests showed that CC significantly improved 1RM in both the bench press (+3.3 ± 7.8%, *p* < 0.001, *d =* 0.26) and back squat (+4.5 ± 5.0%, *p* = 0.009, *d =* 0.35) compared to placebo. CG showed a trend toward a larger 1RM (+2.1 ± 5.0%, *p* = 0.085, *d =* 0.11) in the bench press and a significant improvement in the back squat (+5.0 ± 6.0%, *p* = 0.003, *d =* 0.39) compared to PG (Figure 3). Additionally, a significant main effect of mode was observed for 1RM in both the bench press (*F* = 6.153, *p* = 0.030, *ηp^2^* = 0.29) and back squat (*F* = 5.292, *p* = 0.041, *ηp^2^* = 0.26) (Figure 1). However, post-hoc tests revealed no significant difference in 1RM between CC and CG (*all p* > 0.05) for both exercises (Figure 3). In addition, there was no significant difference in the percent change of 1RM between modes (*p* > 0.05) (Figure 3).

Error bars represent standard deviation.

* p < 0.05 vs. PC, ^#^ p < 0.05 vs. PG.

Abbreviations: CC, caffeine capsule; PC, placebo capsule; CG, caffeinated gum; PG, placebo gum.

### 3.2. Muscular power

**Figure 4.**
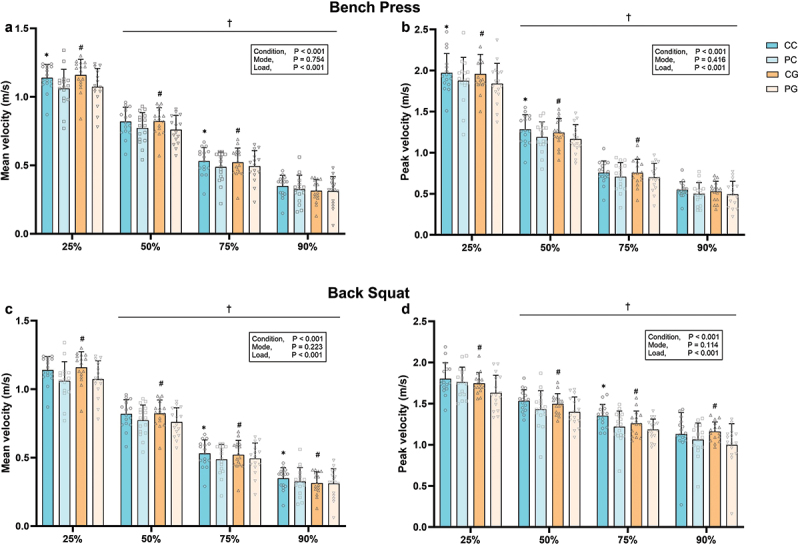
Mean and peak velocity in the bench press (a and b) and back squat (c and d).

A significant main effect of condition was detected for MV in both the bench press (*F* = 18.237, *p* < 0.001, *ηp^2^* = 0.55) and back squat (*F* = 29.719, *p* < 0.001, *ηp^2^* = 0.67) (Figure 4). Post-hoc tests showed that CC significant improved MV in the bench press at 25%1RM (+8.2 ± 8.4%, *p* = 0.002, *d =* 0.64) and 75%1RM (+13.0 ± 19.5%, *p* = 0.050, *d =* 0.41), and in the back squat at 75%1RM (+11.8 ± 14.7%, *p* = 0.002, *d =* 0.67) and 90%1RM (+12.3 ± 12.0%, *p* < 0.001, *d =* 0.64) compared to PC. Additionally, CG significant improved MV in the bench press at 25%1RM (+8.7 ± 9.6%, *p* = 0.002, *d* = 0.68), 50%1RM (+9.0 ± 10.4%, *p* = 0.004, *d* = 0.62) and 75%1RM (+7.0 ± 11.8%, *p* = 0.047, *d* = 0.25), and in the back squat at 25%1RM (+6.7 ± 8.9%, *p* = 0.012, *d* = 0.72), 50%1RM (+8.8 ± 10.4%, *p* = 0.005, *d* = 0.77), 75%1RM (+12.3 ± 13.2%, *p* < 0.001, *d* = 0.66) and 90%1RM (+20.8 ± 20.6%, *p* < 0.001, *d* = 0.86) (Figure 4). However, there was no significant main effect of mode on MV in either the bench press or back squat (all *p* > 0.05) (Figure 4).

Error bars represent standard deviation.

^*^p < 0.05 vs. PC, ^*#*^*p < 0.05 vs. PG*, ^*†*^*p < 0.05 vs. 25%1RM.*

Abbreviations: CC, caffeine capsule; PC, placebo capsule; CG, caffeinated gum; PG, placebo gum.

A significant main effect of condition was detected for PV in both the bench press (*F* = 23.245, *p* < 0.001, *ηp^2^* = 0.61) and back squat (*F* = 59.303, *p* < 0.001, *ηp^2^* = 0.80) (Figure 4). Post-hoc tests showed that CC significant improved PV in the bench press at 25%1RM (+6.1 ± 10.3%, *p = 0.032*, *d =* 0.38), 50%1RM (+8.6 ± 9.7%, *p* = 0.008, *d =* 0.51), and 90%1RM (+10.3 ± 19.4%, *p* = 0.052, *d =* 0.42), and in the back squat at 75%1RM (+14.3 ± 28.8%, *p* = 0.019, *d =* 0.79) compared to PC. Additionally, CG significantly improved PV in the bench press at 25%1RM (+7.0 ± 9.2%, *p* < 0.001, *d* = 0.49), 50%1RM (+7.5 ± 10.0%, *p* = 0.013, *d* = 0.46) and 75%1RM (+9.8 ± 13.5%, *p* = 0.012, *d* = 0.35), and in the back squat at 25%1RM (+8.4 ± 12.0%, *p* = 0.024, *d* = 0.66), 50%1RM (+7.8 ± 10.6%, *p* = 0.014, *d* = 0.62), 75%1RM (+6.7 ± 9.7%, *p* = 0.020, *d* = 0.54) and 90%1RM (+18.2 ± 31.8%, *p* = 0.020, *d* = 0.81) (Figure 4). However, there was no significant main effect of mode on PV in either the bench press or back squat (all *p* > 0.05) (Figure 4).

**Figure 5.**
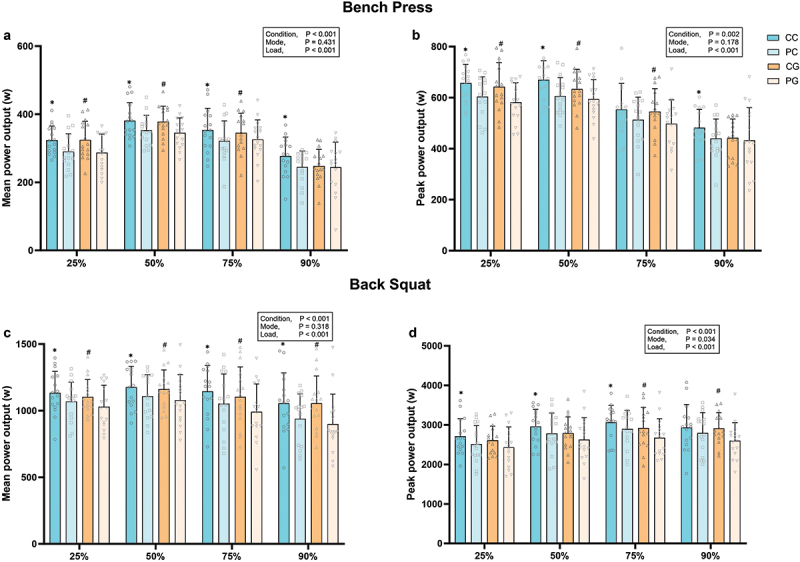
Mean and peak power output in the bench press (a and b) and back squat (c and d).

A significant main effect of condition was detected for MPO in both the bench press (*F* = 18.788, *p* < 0.001, *ηp^2^* = 0.56) and back squat (*F* = 37.227, *p* < 0.001, *ηp^2^* = 0.71) (Figure 5). Post-hoc tests showed that CC significant improved MPO in the bench press at 25%1RM (+12.9 ± 13.4%, *p* = 0.002, *d =* 0.70), 50%1RM (+8.6 ± 13.8%, *p* = 0.033, *d =* 0.58), 75%1RM (+11.9 ± 20.5%, *p* = 0.049, *d =* 0.53) and 90%1RM (+14.5 ± 20.9%, *p* = 0.009, *d =* 0.62), and in the back squat at 25%1RM (+6.6 ± 10.3%, *p* = 0.016, *d =* 0.50), 50%1RM (+7.2 ± 10.0%, *p* = 0.050, *d =* 0.48), 75%1RM (+9.9 ± 10.0%, *p* = 0.011, *d =* 0.52) and 90%1RM (+14.3 ± 14.7%, *p* = 0.001, *d =* 0.72) compared to PC (Figure 5). Additionally, CG significantly improved MPO in the bench press at 25%1RM (+14.4 ± 15.4%, *p* < 0.001, *d* = 0.68), 50%1RM (+10.2 ± 11.6%, *p* < 0.001, *d* = 0.74) and 75%1RM (+7.0 ± 11.8%, *p* = 0.047, *d* = 0.25), and in the back squat at 25%1RM (+8.2 ± 10.7%, *p* = 0.020, *d* = 0.50), 50%1RM (+8.8 ± 10.8%, *p* = 0.013, *d* = 0.47), 75%1RM (+12.4 ± 12.9%, *p* < 0.001, *d* = 0.52), and 90%1RM (+20.0 ± 20.9%, *p* = 0.002, *d* = 0.72) (Figure 5). However, there was no significant main effect of mode on MPO in either the bench press or back squat (all *p* > 0.05) (Figure 5).

Error bars represent standard deviation.

^*^p < 0.05 vs. PC, ^*#*^*p < 0.05 vs. PG*, ^*†*^*p < 0.05 vs. 25%1RM.*

Abbreviations: CC, caffeine capsule; PC, placebo capsule; CG, caffeinated gum; PG, placebo gum.

A significant main effect of condition was detected for PPO in the bench press (*F* 14.669, *p* = 0.002, *ηp^2^* = 0.49) and back squat (*F* = 22.382, *p* < 0.001, *ηp^2^* = 0.60) (Figure 5). Post-hoc tests showed that CC significant improved PPO in the bench press at 25%1RM (+10.1 ± 16.0%, *p* = 0.021, *d =* 0.70), 50%1RM (+11.3 ± 13.6%, *p* = 0.005, *d =* 0.86) and 90%1RM (+11.9 ± 22.1%, *p* = 0.041, *d =* 0.57), and in the back squat at 25%1RM (+9.4 ± 15.6%, *p* = 0.046, *d =* 0.43), 50%1RM (+7.3 ± 8.9%, *p* = 0.009, *d =* 0.37) and 75%1RM (+6.7 ± 10.7%, *p* = 0.024, *d =* 0.38) compared to PC (Figure 5). Additionally, CG significantly improved PPO in the bench press at 25%1RM (+11.2 ± 14.8%, *p* = 0.008, *d* = 0.71), 50%1RM (+7.5 ± 13.7%, *p* = 0.043, *d* = 0.51) and 75%1RM (+11.2 ± 18.3%, *p* = 0.037, *d* = 0.52), and in the back squat at 75%1RM (+10.1 ± 15.1%, *p* = 0.027, *d* = 0.49) and 90%1RM (+13.3 ± 13.2%, *p* = 0.002, *d* = 0.73) (Figure 5). A significant main effect of mode was detected for PPO in the back squat (*F* = 5.413, *p* = 0.034, *ηp^2^* = 0.27), but not for the bench press (*F* = 1.994, *p* = 0.178, *ηp^2^* = 0.11) (Figure 3). However, post-hoc tests revealed no significant difference in PPO between CC and CG (*all p* > 0.05) for the back squat (Figure 5).

There was no significant difference in the percent change of muscular power (MV, PV, MPO and PPO) between modes (all *p* > 0.05) (Table 1).

**Table 1.** Percentage improvement (%) in muscular power during bench press and back squat exercises.

Indicator	25%1RM			50%1RM			75%1RM			90%1RM			ANOVA *p* value (*pη2*)		
	Capsule	Gum	Sig	Capsule	Gum	Sig	Capsule	Gum	Sig	Capsule	Gum	Sig	Mode	Load	Mode × load
**bench press**															
mean velocity	8.2 ± 8.4	8.7 ± 9.6	0.888	7.0 ± 12.2	9.0 ± 10.4	0.563	13.0 ± 19.5	7.0 ± 11.8	0.176	11.7 ± 22.3	10.5 ± 35.9	0.908	0.739(0.01)	0.953(0.02)	0.792(0.02)
peak velocity	6.1 ± 10.3	7.0 ± 9.2	0.272	8.6 ± 11.0	7.5 ± 9.9	0.097	10.3 ± 19.4	9.8 ± 13.5	0.327	14.9 ± 26.2	15.2 ± 34.1	0.372	0.968(0.00)	0.370(0.07)	0.995(0.00)
mean power output	12.9 ± 13.4	14.6 ± 15.4	0.756	8.6 ± 13.8	10.2 ± 11.6	0.672	11.9 ± 20.5	6.6 ± 11.7	0.26	14.5 ± 19.9	11.3 ± 38.9	0.781	0.743(0.01)	0.611(0.04)	0.838(0.02)
peak power output	10.1 ± 16.0	11.2 ± 14.8	0.831	11.3 ± 13.6	7.5 ± 13.7	0.393	10.8 ± 26.6	11.3 ± 18.3	0.919	11.9 ± 22.1	10.0 ± 33.2	0.839	0.804(0.00)	0.991(0.03)	0.925(0.01)
**back squat**															
mean velocity	3.0 ± 7.5	6.7 ± 8.9	0.171	8.8 ± 10.4	8.8 ± 10.4	0.181	11.8 ± 14.7	12.3 ± 13.2	0.886	12.3 ± 12.0	20.6 ± 20.9	0.135	0.137(0.14)	0.001(0.34)	0.393(0.06)
peak velocity	2.7 ± 9.7	8.4 ± 12.0	0.107	9.8 ± 22.3	7.8 ± 10.6	0.799	14.3 ± 28.8	6.7 ± 9.4	0.377	11.4 ± 44.7	10.3 ± 10.4	0.932	0.866(0.00)	0.621(0.04)	0.445(0.06)
mean power output	6.4 ± 10.3	8.2 ± 10.7	0.613	6.9 ± 11.8	8.8 ± 10.8	0.619	10.6 ± 16.5	12.4 ± 12.9	0.655	12.6 ± 12.6	20.0 ± 20.1	0.179	0.310(0.07)	0.017(0.20)	0.526(0.05)
peak power output	9.4 ± 15.6	9.5 ± 14.9	0.972	7.3 ± 8.9	8.3 ± 16.1	0.839	6.7 ± 10.7	10.1 ± 15.1	0.511	5.3 ± 12.2	13.3 ± 13.3	0.095	0390(0.05)	0.941(0.009)	0.428(0.059)

### 3.3. Perceptual responses

During maximal strength tests, RPE and perceived pain did not differ between conditions for both the bench press and back squat (all *p* > 0.05) (S1).

During muscular power tests, RPE did not differ between conditions (S2). However, a significant main effect of condition was detected for perceived pain in the back squat (*F* = 4.727, *p* = 0.046, *ηp^2^* = 0.24), but not for the bench press (*F* = 4.131, *p* = 0.064, *ηp^2^* = 0.22) (S2). Post-hoc tests revealed no significant differences in perceived pain between conditions (all *p* > 0.05) for the back squat (S2).

### 3.4. Assessment of blinding and side effects

As indicated by BBI values, blinding was effective both immediately following ingestion (CC: -0.07 [95%CI: −0.24–0.11]; PC: 0.00 [95% CI: −0.21–0.21]; CG: 0.19 [95%CI: −0.14–0.52]; PG: −0.13 [95% CI: −0.37–0.12]) and post-exercise (CC: 0.13 [95%CI: −0.12–0.37]; PC: 0.06 [95% CI: −0.21–0.33]; CG: 0.27 [95%CI: −0.13–0.66]; PG: 0.13 [95% CI: −0.20–0.45]) (Table 2).

**Table 2.** The number (frequency) of participants who reported side effects immediately later and 24 hours (h) later, out of 16 participants.

Side effect	CAF				PLA			
	Capsule		Gum		Capsule		Gum	
	+0 h	+24 h	+0 h	+24 h	+0 h	+24 h	+0 h	+24 h
Muscle soreness	5 (31.25%)	10 (62.5%)*	6 (37.5%)	8 (50%)	5 (31.25%)	4 (25%)	6 (37.5%)	7 (43.75%)
Increased urine output	8 (50%)*	4 (25%)	6 (37.5%)	6 (37.5%)^#^	2 (12.5%)	2 (12.5%)	2 (12.5%)	0 (0%)
Tachycardia and heart palpitations	5 (31.25%)*	4 (25%)^*,$^	5 (31.25%)^#^	0 (0%)	0 (0%)	0 (0%)	1 (6.25)	1 (6.25%)
Anxiety or nervousness	3 (18.75%)	1 (6.25)	2 (12.5%)	1 (6.25%)	0 (0%)	0 (0%)	1 (6.25%)	0 (0%)
Headache	0 (0%)	0 (0%)	0 (0%)	0 (0%)	0 (0%)	0 (0%)	0 (0%)	0 (0%)
Gastrointestinal problems	6 (37.5%)*,^$^	7 (43.75%)*	2 (12.5%)	3 (18.75%)	1 (6.25%)	2 (12.5%)	0 (0%)	0 (0%)
Insomnia	–	5 (31.25%)	–	7 (43.75%)^#^	–	1 (6.25)	–	2 (12.5%)
Increased vigor/activeness	9 (56.25%)*	3 (18.75%)	14 (87.5%)^#^	5 (31.25%)!	3 (18.75%)	2 (12.5%)	4 (25%)	4 (25%)
Perception of performance improvement	7 (43.75%)^$^	–	14 (87.5%)^#^	–	3 (18.75%)	–	2 (12.5%)	–

*p < 0.05 vs. PC, ^#^*p* < 0.05 vs. PG, ^$^*p* < 0.05 vs. CG, ^!^*p* < 0.05 vs. immediately later.

Immediately post-exercise CC significantly increased the incidence of *urine output* (50% vs. 12.5%, *p* < 0.01), *Tachycardia and heart palpitations* (31.2% vs. 12.5%, *p* = 0.011), *Gastrointestinal problems* (37.5% vs. 0%, *p* = 0.011), and *vigor/activeness* (56.2% vs. 6.2%, *p* = 0.030) compared to PC (Table 2). CG significantly increased the incidence of *Tachycardia* and *heart palpitations* (31.2% *vs*. 0%, *p* = 0.041), *vigor/activeness* (87.5% vs. 18.8%, *p* < 0.001), and *Perception of performance improvement* (87.5% vs. 18.8%, *p* < 0.001) compared to PG (Table 2). In addition, CC led to a higher incidence of *Gastrointestinal problems* (37.5% vs. 12.5%, *p* = 0.041) than CG. However, CG resulted the higher incidence of *Perception of performance improvement* (87.5% vs. 43.7%, *p* = 0.010) than CC (Table 2).

24 hours post-exercise, CC significantly increased the incidence of *Muscle soreness* (62.5% vs. 25.0%, *p* = 0.016), *Tachycardia and heart palpitations* (25% vs. 0%, *p* = 0.011), *Gastrointestinal problems* (43.7% vs. 12.5%, *p* = 0.021) compared to PC (Table 2). CG significantly increased the incidence of *Increased urine output* (37.5% vs. 0%, *p* < 0.001) and *Insomnia* (43.7% vs. 12.5%, *p* = 0.038) compared to PG (Table 2). In addition, CC led to the higher incidence of *Tachycardia* and *heart palpitations* (25% vs. 0%, *p* = 0.011) than CG.

For CC, *muscle soreness* showed a trend toward a higher incidence 24 hours post-exercise compared to immediately post-exercise (31.3% vs. 62.5%, *p* = 0.063). For CG, the incidence of *increased vigor/activeness* significantly decreased 24-h post-exercise (87.5% vs. 31.3%, *p* = 0.004), while *tachycardia and heart* palpitations trended toward a lower incidence (31.3% vs. 0%, *p* = 0.063).

## 4. Discussion

Our primary finding was that caffeine (4 mg/kg) significantly improved maximal strength and muscular power during the bench press and back squat in resistance-trained men, regardless of whether it was administered via capsule or chewing gum. As hypothesized, CG led to fewer instances of *gastrointestinal discomfort* immediately post-exercise and *tachycardia/heart palpitations* 24 hours later compared to CC. While performance benefits did not significantly differ between methods, CG may be preferred due to its comparable ergogenic effects and fewer side effects.

Our study reported that caffeine delivered via capsules and chewing gum produced similar ergogenic effects on 1RM, MV, PV, MPO and PPO during the bench press and back squat in resistance-trained men (Figures 1–3). This aligns with previous research by the same group, which found no performance differences across caffeine (3–4.5 mg/kg) delivery methods (e.g. gum, tablet, and strip) when administered 15 min before a 5‑km running time trial, compared to placebo capsule [25,35]. However, those studies had limitations in caffeine timing and placebo design. Specifically, administrating all caffeine forms 15 min before exercise overlooks their distinct pharmacokinetics [25,35]. Indeed, caffeine delivered via chewing gum reaches effective serum concentrations within 5–10 min [18], while traditional modes (e.g. tablets and capsules) require 45–60 min to peak [16]. Furthermore, using capsules as a placebo for all delivery methods (such as gum, tablets, and strips) may have compromised blinding [25]. Our study addressed these issues and confirmed that caffeine, whether administered in chewing gum or capsules, produced comparable benefits on maximal strength and muscular power during bench press and back squat exercises. Although these findings contradict our initial hypothesis, they are understandable. While CG bypasses first-pass metabolism, it may not increase peak serum caffeine concentrations. A previous study supports this notion, showing comparable peak serum caffeine concentrations after ingesting either CG or CC (200 mg) [16]. However, since our study did not measure blood caffeine concentration, future research should compare serum caffeine concentrations from different doses of capsules and gum to validate this speculation.

There were no significant differences between CG and CC in percentage improvements for 1RM (+2.1–5.0% vs. +3.3–4.5%) and MV (+6.7–20.6% vs. +3.0–13.0%) (Table 1). Recent evidence further supports our findings, demonstrating that CC and CG (3–4.5 mg/kg) produce comparable effects on the muscular power and strength of knee extensors during isokinetic and isometric contractions in strength-trained males [4]. However, indirect comparisons indicate that CG may induce greater improvements in maximal strength (+5.0–6.8% vs. +1.3–4.5%) and muscular power (+5.7–9.1% vs. +1.0–5.6%) in studies recruiting males with low-to-mild caffeine consumption ( < 3 mg/kg/day) [20,21], compared to CC studies that included both sexes [14] or participants with high caffeine consumption ( > 6 mg/kg/day) [12,15]. The relatively smaller percentage improvements observed with CC may be attributed to the inclusion of female participants and regular caffeine users. Hormonal fluctuations during the menstrual cycle may impair caffeine metabolism and reduce its ergogenic effects in females [36], while habitual caffeine intake (≥3 mg/kg/day) may increase tolerance, thereby diminishing caffeine’s performance-enhancing effects [37]. Taken together, our results and previous findings indicate that CG and CC produce similar ergogenic effects on maximal strength and muscular power during bench press and back squat in resistance-trained men with naïve-to-mild caffeine consumption habits.

Our results showed that caffeine significantly enhanced 1RM, MV, PV, MPO and PPO during bench press and back squat in resistance-trained men, regardless of the absorption method (Figures 1–3). These observations align with recent meta-analyses demonstrating that caffeine (1–9 mg/kg) boost maximal strength and muscular power in both upper and lower limbs during resistance exercises [38,39]. Mechanistically, caffeine is thought to reduce perceived fatigue and pain, supporting its role in enhancing strength [7,40]. Our results further support this mechanism, demonstrating that caffeine enhanced back squat 1RM without exaggerating RPE and pain perception (S1–2). Additionally, caffeine may augment central drive by increasing motor unit recruitment and firing frequency, and improve muscle contractility through upregulated sodium-potassium and calcium pumps [16,41]. However, further studies using percutaneous nerve stimulation are necessary to shed more light on these potential mechanisms. Notably, the bitter taste of CG may stimulate brain regions responsible for motor control and arousal, potentially improving muscle strength [42,43].

This study is the first to compare side effects between different caffeine delivery methods and found that, compared to CC, CG resulted in fewer instances of *gastrointestinal discomfort* immediately after exercise (Table 2). This may stem from caffeine in chewing gum being absorbed primarily through the oral mucosa, reducing gastrointestinal irritation during intense activity [18]. Additionally, 24 hours post-exercise, CG was linked to a reduced incidence of *tachycardia or heart palpitations* compared to CC (Table 2) and the incidence of this showed a trend toward lower versus immediately after exercise. This may be attributed to the faster absorption of caffeine from gum [16], leading to earlier metabolic clearance and reduced cardiovascular solicitation post-exercise. In support, previous research found that participants consuming caffeine (3–4.5 mg/kg) via gum had a significantly higher urinary paraxanthine-to-caffeine ratio compared to those using tablets [35]. Notably, genetic variation may influence caffeine metabolism and its side effects [8]. Individuals carrying the AA genotype metabolize caffeine faster than those with CC or AC genotypes [2,44], though further research is required to confirm this. Furthermore, 24 hours post-exercise, CG showed a trend toward reduced incidence of tachycardia and heart palpitations compared to immediately post-exercise, while CC revealed an upward trend in the incidence of muscle soreness. This suggests that CG may impose a lower physiological burden during post-competition recovery, making it particularly suitable for athletes with short recovery periods between competitions.

## 5. Limitations and additional considerations

Several limitations should be considered when interpreting these findings. First, while our double-blind, placebo controlled designed allowed for separate assessment of capsules and chewing gum, the interaction between different caffeine absorption methods (i.e. strips, gels, mouthwash) remains unclear. Combining methods could have minimal or even negative effects due to uncertainties around optimal dosing and timing. Understanding the mechanisms behind each method could reveal potential synergies that enhance resistance exercise performance. Our study focused on mechanical output variables; however, incorporating biochemical variables (e.g. blood caffeine, paraxanthine, and heart rate variability) and neuromechanical data (e.g. surface electromyography, 3D motion capture) would provide deeper insights into how the administration mode influences the magnitude of these effects. Additionally, as we assessed performance with low-volume resistance exercises, we cannot determine if caffeine affects outcomes in higher-volume training or different set arrangements (e.g. traditional vs. cluster sets). Finally, since our study included only young, healthy, resistance-trained men, the findings may not be applicable to other populations such as sedentary individuals, injured athletes, females, adolescents, or older adults. Future research should include more diverse participant groups to enhance the robustness and applicability of the results.

## 6. Conclusion

Caffeinated gum (4 mg/kg) produced ergogenic effects comparable to capsules in enhancing maximal strength and muscular power during bench press and back squat exercises, with fewer side effects in resistance-trained men. These findings offer practical guidance for athletes and coaches to develop time-efficient caffeine supplementation strategies to improve resistance exercise performance while minimizing side effect risks. Nevertheless, individual factors such as sex, genetics, and training status should be considered when personalizing supplementation regimens.

## Data Availability

Data are available from the corresponding author upon reasonable request.
